# Identification of Distinct Molecular Subtypes of Endometrioid Adenocarcinoma

**DOI:** 10.3389/fgene.2021.568779

**Published:** 2021-07-21

**Authors:** Jia Lei, Shuping Yan, Xiangqian Guo, Fengling Wang, Guosen Zhang, Quancheng Kan, Ruixia Guo

**Affiliations:** ^1^Department of Gynecologic Oncology, The First Affiliated Hospital of Zhengzhou University, Zhengzhou, China; ^2^Joint National Laboratory for Antibody Drug Engineering, Cell Signal Transduction Laboratory, Department of Preventive Medicine, School of Basic Medical Sciences, Institute of Biomedical Informatics, Henan University, Kaifeng, China

**Keywords:** endometrioid adenocarcinoma, molecular subtype, molecular signature, gene expression pattern, subtype-specific treatment

## Abstract

Endometrial carcinoma (EC) is one of the most common gynecological cancers worldwide. Endometrioid adenocarcinoma (EAC) is the major form of EC, accounting for 75–80% of cases. Currently, there is no molecular classification system for EAC, so there are no corresponding targeted treatments. In this study, we identified two distinct molecular subtypes of EAC with different gene expression patterns and clinicopathologic characteristics. Subtype I EAC cases, accounting for the majority of cases (56%), were associated with an earlier stage, a more well-differentiated grade, a lower tumor invasion rate, and a more favorable prognosis, and the median tumor necrosis percent (15%) was also significantly higher in subtype I EAC. In contrast, subtype II EAC represents high-grade EAC, with a higher tumor invasion rate and tumor weight. The up-regulated genes in subtype I EAC were associated with the immune response, defense response, cell motion, and cell motility pathway, whereas the up-regulated genes in subtype II EAC were associated with the cell cycle, DNA replication, and RNA processing pathways. Additionally, we identified three potential subtype-specific biomarkers, comprising *MDM2* (MDM2 proto-oncogene) for subtype I, and *MSH2* (mutS homolog 2) and *MSH6* (mutS homolog 6) for subtype II.

## Introduction

Endometrial carcinoma (EC) is one of the most common gynecological cancers in China (Yang et al., 2005) and the fourth most common pelvic malignancy in women around the world (Jemal et al., 2007). ECs can be divided into two pathogenetic types. Type I ECs are generally endometrioid adenocarcinomas (EACs), accounting for the majority of ECs (75–80%), and they are associated with unopposed estrogen stimulation, higher estrogen receptor expression, and a more favorable prognosis. In contrast, type II ECs are mostly serous carcinomas and are associated with poor differentiation, deep myometrial invasion, metastasis to pelvic lymph nodes, lower rates of progestogen sensitivity, and poorer prognosis (Bokhman, 1983; Emons et al., 2000; Sherman, 2000; Hecht and Mutter, 2006). However, this classification has limitations. For example, 20% of EAC cases are high-grade carcinomas and excluded from type I (Bokhman, 1983; Zannoni et al., 2010; Brinton et al., 2013). Additionally, some EAC patients with positive estrogen receptor status do not respond to anti-estrogen therapy.

In the past few decades, a number of tumor types (breast cancer, bladder cancer, colon cancer, kidney cancer, ovarian cancer, leiomyosarcoma, etc.) have been successfully stratified into molecular subtypes (Sorlie et al., 2001; Lapointe et al., 2004; Verhaak et al., 2010; Youssef et al., 2011; Marisa et al., 2013; Choi et al., 2014; Damrauer et al., 2014; Guo et al., 2015; Ceccarelli et al., 2016). This has improved our understanding of the tumorigenesis and progression mechanisms and has also provided us with opportunities to develop more effective therapeutic methods. Therefore, it is of clinical importance for us to identify the molecular subtypes of EAC.

Recently, several groups have identified molecular alterations of several important genes in EC (Cancer Genome Atlas Research Network et al., 2011; Travaglino et al., 2020a,b,c). The Cancer Genome Atlas (TCGA) subdivided EC into four molecular subgroups with a potential prognostic significance: POLE-mutated/ultramutated (POLEmt), microsatellite-instable/hypermutated (MSI), copy-number-low/p53-wild-type (p53wt), and copy-number-high/p53-mutated (p53mt). Two molecular classifiers including *Trans*PORTEC (Stelloo et al., 2015) and ProMisE (Talhouk et al., 2015, 2017) were subsequently established for translational research using targeted sequencing and immunohistochemistry. However, the following studies revealed that the prevalence of the TCGA subgroups was not in accordance with the prognostic value of FIGO grade, histotype of EC (Raffone et al., 2019; Travaglino et al., 2020d,e). Therefore, more studies need to be carried out to recognize the molecular subtypes for EAC, and the successful identification of EAC molecular subtypes and the diagnostic markers for these subtypes will also lay the foundation for the development of targeted therapies for EAC.

In this study, we performed gene expression-based molecular subtyping though consensus clustering analyses, and identified two molecular subtypes for EAC in two independent EAC cohorts. Furthermore, each of these subtypes has distinct expression pattern, subtype-specific biomarkers and potential therapeutic targets.

## Materials and Methods

### Determination and Validation of Subtype in Two EAC Cohorts

Two independent EAC gene expression datasets were collected from TCGA and the Gene Expression Omnibus (GEO) database (GSE32507). Then these expression data was filtered based on standard deviation according to our previously studies An et al. (2017a, b). To determine the molecular subtypes of EAC, consensus clustering (Consensus Clustering Plus R package) (Wilkerson and Hayes, 2010) was employed on TCGA (Discovery cohort) and GSE32507 (Validation cohort) independently using the following parameters: Distance: 1—Pearson correlation; gene and sample resampling: 80%; and maximum evaluated k: 12. Silhouette widths (cluster R package) were then computed to assess the accuracy of the classification determined by Consensus Clustering Plus.

### Measurement of the Reproducibility of EAC Subtypes Among the Two Independent Cohorts

Subclass mapping (Hoshida et al., 2007) of the GenePattern was used to determine the reproducibility of EAC molecular subtypes between TCGA datasets and GSE32507. The SubMap was run with parameters of “num. marker. genes = 300, num. perm = 1000 and num. perm. fisher = 1000.”

### Subtype Specific Gene Expression Patterns Analysis

Differentially expressed genes (DEG) between the two EAC subtypes were analyzed by SAM-seq significance analysis with a false discovery rate of (FDR) of 0.05. Subsequently, functional annotation of subtype specific high expressed gene in each subtype was performed using DAVID Bioinformatics Resources (version 6.7)^1^, and GSEA (Subramanian et al., 2005). DEGs between each subtype were clustering by Cluster 3.0 (de Hoon et al., 2004.) and visualized by TreeView (Saldanha, 2004). The TARGET V2 database^2^ was used to identify potential therapeutic targets in each EAC subtype.

### Immunohistochemical Analysis

To identify subtype-specific biomarkers, tissues from 237 primary EAC cases were collected from 2011–2017 at the First Affiliated Hospital of Zhengzhou University and subjected to tissue microarray (TMA). Ethics approval was granted by the hospital’s institutional review board. Antibodies against MSH2 (1:200; D24b5; CST, United States), MSH6 (1:500; 3E1; CST, United States), and MDM2 (1:500; ab38618; Abcam, China) were used in the immunohistochemical analysis, as these corresponded to the three top-ranked subtype-specific genes for which high-quality commercial antibodies were available based on the SAM-seq results. The immunohistochemistry (IHC) was performed according to Wang et al. (2019). In brief, the TMA sections were baked in an oven for 1 h, deparaffinized, and rehydrated with xylene and graded alcohols. After blocking by 3% H_2_O_2_ for 15-min incubation, the sections were boiled in antigen retrieval buffer. Then, the TMAs were incubated in primary antibodies and HRP-conjugated secondary antibodies (Boster). After developing with diaminobenzidine, the sections were counterstained with hematoxylin and dehydrated in graded alcohols and xylene. Abcam, CST) were used. The immunohistochemical staining was graded as follows: 0, no staining; 1, weak staining (10% of cells); 2, moderate staining (10–30%); and 3, strong staining (> 30%).

### Statistical Analysis

The statistical analysis was performed using Fisher’s exact test and the chi-square test in GraphPad software. Kaplan–Meier curves and Log-rank (Mantel–Cox) test was performed to compute the statistical significance of survival time between different subtypes by using GraphPad software, where *P*-value < 0.05 was considered statistically significant.

## Results

### Consensus Clustering Revealed Two Molecular Subtypes of EAC

**FIGURE 1:**
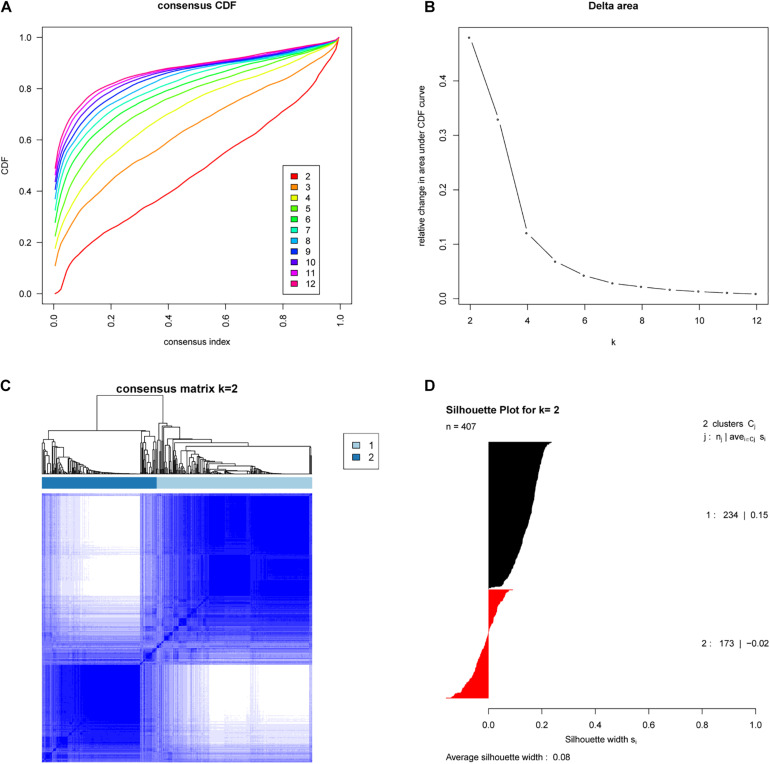
Identification of two molecular subtypes of EAC in the TCGA cohort (Discovery cohort). **(A)** Empirical cumulative distribution plot determines the optimal number of EAC molecular subtypes. **(B)** Relative increase in the area under the CDF curve along with increasing assumed number of molecular subtypes. **(C)** Consensus Clustering matrix shows two molecular subtypes of EAC in TCGA. **(D)** Silhouette analysis of TCGA cohort using assignments from Consensus Clustering.

We analyzed the expression profiles of 407 EAC cases from TCGA by consensus clustering, which uses data resampling to assess the cluster stability. The result showed that two subtypes were optimal. As shown in Figure 1, when two subtypes were assumed, the relative change in the area under the cumulative distribution function (CDF) curve was the greatest. To confirm the subtype assignment based on consensus clustering, a silhouette analysis was then performed, which revealed that all subtype I cases had a positive value while one subtype II case had a negative value. The negative silhouette values were considered unreasonable and were classified as “other,” which is not belong to subtype I or subtype II. Only cases with positive silhouette values were used in the further analyses (An et al., 2017a,b).

### Validation of Subtypes in an Independent Cohort

**FIGURE 2:**
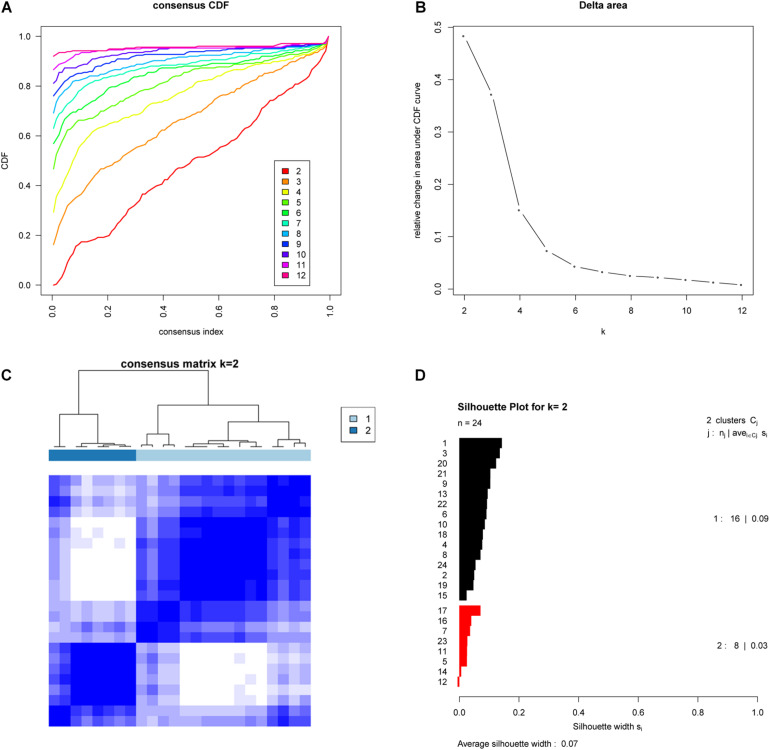
Validation of molecular subtypes of EAC in the GSE32507 cohort (Validation cohort). Empirical cumulative distribution plot **(A)** and Relative increase in the area under the CDF curve **(B)** in molecular subtypes of GSE32507; Consensus Clustering matrix **(C)** and Silhouette analysis **(D)** of the two subtype of GSE32507.

To further investigate whether these subtypes are specific to this EAC cohort or a result of the methodology employed, we examined whether the two EAC molecular subtypes could also be found in an independent cohort. To do this we examined publicly available gene expression data (based on 24 EAC cases) from the GEO database (GSE32507), and performed the same subtyping analysis as for the TCGA cohort. Remarkably, this also showed that two molecular subtypes were optimal (Figure 2).

**FIGURE 3:**
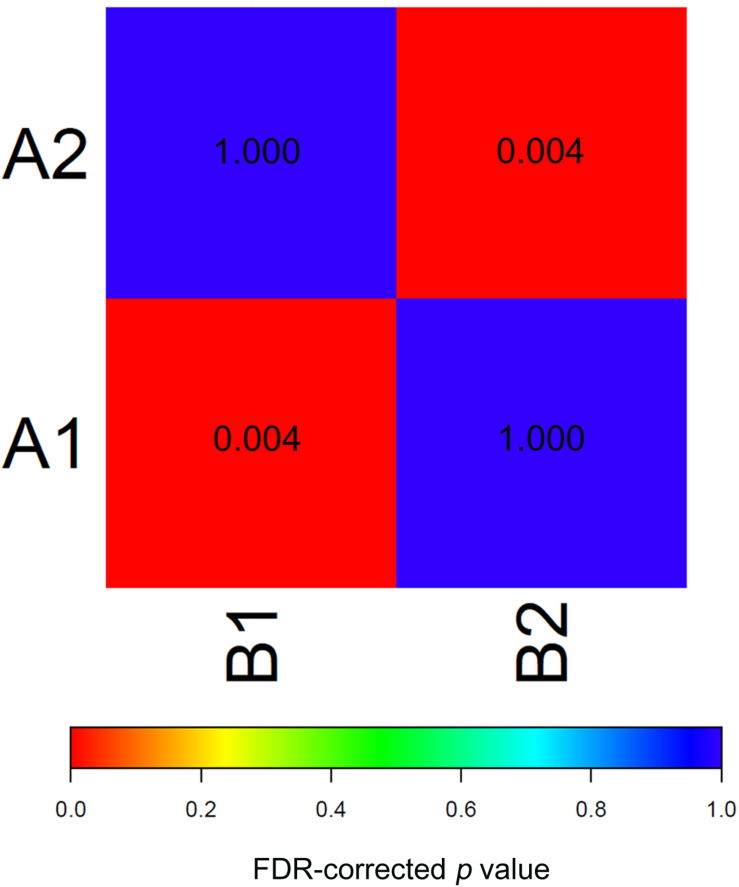
SubMap association matrix among TCGA (subtype A1 and subtype A2), GSE32507 (subtype B1 and subtype B2), *P*-value is corrected with FDR.

To test the reproducibility of the two EAC molecular subtypes between the GEO and TCGA datasets, we performed subclass mapping (SubMap) between the two datasets, using all cases with positive silhouette values. The results showed that TCGA subtypes B1 and B2 were significantly correlated with GEO subtypes A1 and A2, suggesting that the two molecular subtypes were significantly reproduced in the two different cohorts (Figure 3).

### Molecular Subtypes and Their Correlation to Clinical Features in TCGA

TCGA data included detailed clinical information for the EAC cases, so we determined the associations between the EAC molecular subtypes and the clinical features in using TCGA data. As shown in Table 1, 68, 8, 16, and 3% of the EACs were stage I, II, III, and IV, respectively. Stage I cases were more common in subtype I (178 out of 230) than subtype II (39 out of 67; χ^2^-test, *P* = 0.003). In addition, we found that more than half of the subtype I cases were grade I–II, while most of the subtype II cases were grade III. The median tumor invasion percent was significantly lower for subtype I (34%) than subtype II (53%; *P* < 0.0001). Additionally, the median tumor necrosis percent was significantly higher for subtype I (30%) than subtype II (10%; *P* = 0.0001). Most importantly, the subtype I patients were more likely to be tumor free after treatment (205 out of 225) than the subtype II patients (51 out of 61; *P* = 0.0416). Furthermore, Kaplan–Meier analysis indicated that the subtype II patients had worse 3 years overall survival than the subtype I patients [Logrank *p* = 0.0484; HR (95%CI) = 2.550 (1.007–6.459)].

**TABLE 1:** Clinicopathologic characteristics of the TCGA EAC cohort.

**Characteristic**	**Patients n (%)**	**Subtype I**	**Subtype II**	**Other**	***P-*value**
**Age (year)**					0.3538
Median	78	61	63	64	
Range	31–90	31–89	33–90	40–90	
**Stage**					0.0003
I	280 (68%)	178	39	63	
II	34 (8%)	21	5	8	
III	69 (16%)	28	17	24	
IV	14 (3%)	3	6	5	
Unknown	10 (1%)	3	3	4	
**Histologic grade**					<0.0001
G1	96 (24%)	87	0	9	
G2	115 (28%)	81	10	24	
G3	186 (46%)	62	57	67	
Unknown	10 (2%)	3	3	4	
**Tumor invasion percent**					<0.0001
Median	140	34	53	140	
Range	0–280	0–100	1.03–100	0–280	
**Percent necrosis TOP**					0.0001
Median	10	15	5	5	
Range	0–30	0–30	0–30	0–25	
**Cancer status**					<0.0416
Tumor free	337 (79%)	205	50	82	
With tumor	44 (10%)	20	11	13	
Unknown	48 (11%)	9	30	9	
**Weight**					0.0153
Median	74	92	107	86	
Range	44–209	48–209	45–142	44–146	
**New tumor event**					0.0915
No	279 (69%)	134	38	67	
Yes	37 (9%)	17	10	10	
Unknown	91 (22%)	42	22	27	
**Primary therapy outcome**					0.4358
Complete remission	309 (76%)	192	45	79	
Partial remission	7 (2%)	3	2	0	
Progressive disease	11 (3%)	4	2	5	
Stable disease	6 (1%)	4	2	0	
Unknown	74 (18%)	31	20	23	
**Vital status**					0.2944
Living	361 (89%)	212	59	90	
Deceased	36 (9%)	18	8	10	
Unknown	10 (2%)	3	3	3	
Follow up time (days)					0.4461
Median	944	1020	744	873	
Range	2–6,859	5–5,651	2–6,859	6–3,067	

### Functional Analysis of EAC Subtype-Specific Genes

**FIGURE 4:**
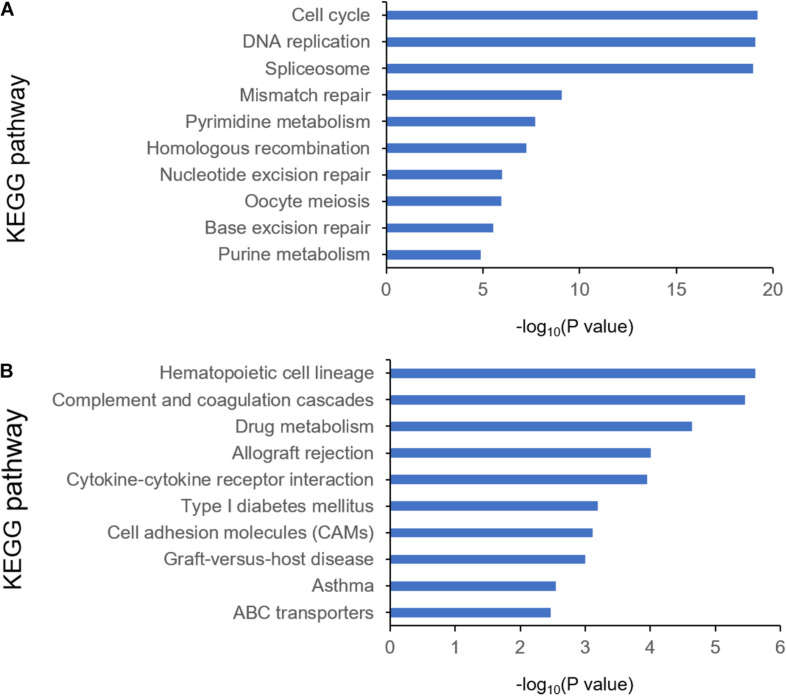
KEGG pathway by analyzing overexpressed genes in each EAC subtype I **(A)** and subtype II **(B)**.

**FIGURE 5:**
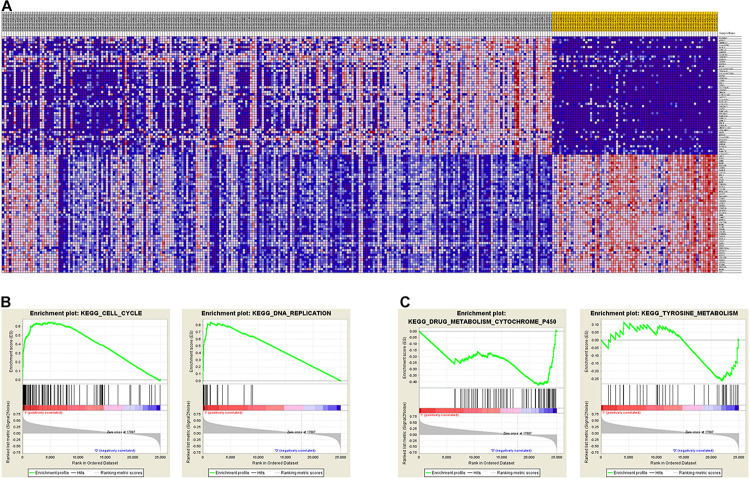
GSEA shows different gene expression signatures in distinct EAC molecular subtypes. **(A)** Different gene expression patterns in subtype I and subtype II, overexpressed genes shown in Red and downexpressed genes shown in blue. **(B)** GSEA demonstrated cell cycle and DNA replication pathways in subtype I. **(C)** GSEA showed enhanced activity of ribosome and drug metabolism cytochrome P450 and tyrosine metabolism in subtype II.

To identify the differentially expressed genes between the two EAC subtypes, SAM-seq (two-class comparison) was used, which identified 13,023 differentially expressed genes. Though Gene Ontology (GO) enrich analysis, up-regulated genes in subtype I EAC were found to be involved in the immune response, defense response, cell motion, and cell motility pathways. In contrast, genes up-regulated in subtype II EAC were found to be involved in the cell cycle, cell division, DNA replication, and RNA processing (Figure 4). The GSEA showed that the up-regulated genes in subtype I were enriched in drug metabolism–cytochrome-P450 and tyrosine metabolism, while the up-regulated genes in subtype II were enriched in DNA replication and cell cycle (Figure 5).

### Identification of EAC Subtype-Specific Biomarkers

**FIGURE 6:**
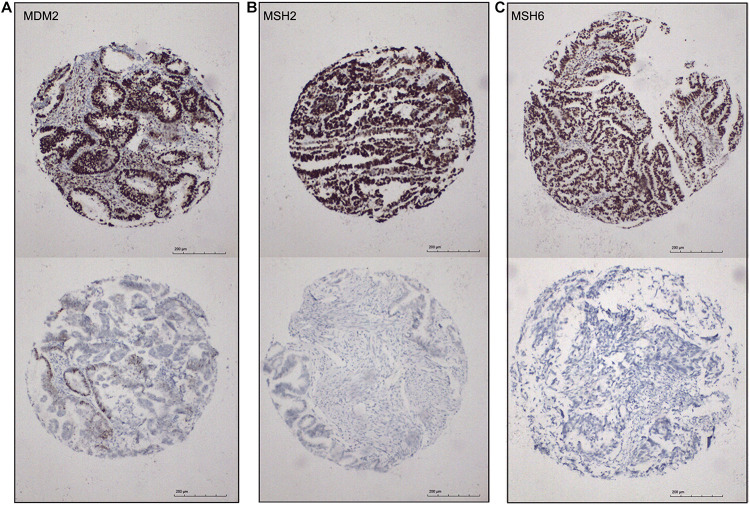
Immunohistochemical markers for subtype I and subtype II ECA. **(A–C)** Representative staining for MDM2, MSH2, and MSH6, respectively (positive, score 3; negative, score 0), 100×.

Identification of biomarkers for molecular subtypes of EAC will provide new insights to the future diagnosis of these subtypes and guide the subtype-specific and effective therapies. Based on the SAM-seq results, we found three top-ranked subtype-specific genes (*MDM2*, *MSH2*, and *MSH6*) that could help to distinguish between the molecular subtypes and for which high-quality commercial antibodies were available for immunohistochemistry staining. *MDM2* was up-regulated in subtype I EAC, while *MSH2* and *MSH6* were up-regulated in subtype II. The immunohistochemistry results showed that 54% (100 out of 184) of the EAC cases were positive for *MSH2*, 51% (96 out of 185) were positive for *MSH6*, and 41% (78 out of 187) were positive for *MDM2* (Figure 6). Both *MSH2* expression (*r* = −0.278, *P* < 0.001) and *MSH6* expression (*r* = −0.220, *P* = 0.003) were significantly negatively correlated with *MDM2* (Figure 6).

### Potential Therapeutic Applications Regarding the EAC Molecular Subtypes

To provide insights into potential targeted therapy for EAC, we compared the top-ranked up-regulated genes in each of the EAC subtypes with genes in the TARGET database, which contains genes that are altered in cancer and are directly linked to clinical effects. This database has been frequently used as a tool to facilitate the identification of therapeutic targets (An et al., 2017a,b). The comparison results showed that five target genes (*AR*, *RARA*, *ESR*1, *ERG*, and *PIK3R1*) were up-regulated in subtype I EAC, while 11 target genes (*AURKA*, *CDKN1B*, *CCNE1*, *CDK4*, *EZH2*, *CRKL*, and *BRCA1*) were up-regulated in subtype II EAC. All 16 genes encode proteins that have specific inhibitors available, and these proteins may act as key therapeutic targets in clinical trials (Table 2).

**TABLE 2:** Potential target genes enriched in each molecular subtype.

**Gene over-expressed**		**Examples of potential therapeutic agents**
Subtype I	AR	Androgen deprivation, Enzalutamide
	RARA	ATRA, Arsenic
	ESR1	Hormonal therapy
	ERG	PARP inhibitor
	PIK3R1	PI3K/AKT/MTOR inhibitors
	AURKA	AURKA Inhibitors
	CDKN1B	CDK inhibitors
	CCNE1	CDK2 inhibitor
	CDK4	CDK4/6 inhibitors
	EZH2	EZH2 inhibitors
Subtype II	CRKL	Gefitinib, Erlotinib, EGFR inhibitors, Vemurafenib, Dabrafenib, RAF inhibitors, Dasatinib, SRC inhibitors
	BRCA2	PARP inhibitor
	BRCA1	PARP inhibitor
	PIK3CA	PI3K/AKT/MTOR inhibitors
	XPO1	SINE agents
	NRAS	Vemurafenib, Dabrafenib, RAF inhibitors

## Discussion

EC is a common cancer in women. It has been estimated that there will be 65,620 new EC cases and 12,590 deaths from EC in 2020 in the United States (Siegel et al., 2020). EAC, which is the most common subclass of EC, mainly occurs in postmenopausal women. However, the survival rate is still very low (<20%) for advanced-stage and recurrent cases of EAC (Brasseur et al., 2017). Therefore, novel approaches for identifying high-risk EAC patients are needed to guide clinical management and improve outcomes.

In the current study, we identified two clinically relevant molecular subtypes of EAC with subtype-specific gene expression patterns, and provided subtype-specific biomarkers and potential therapeutic applications regarding the EAC molecular subtypes. In previously study, TCGA has identified four subtypes of EC using genomic data, which has generated important advances into the genomic characterization of EC. However, the analysis method used in TCGA was too expensive to apply in the current clinical practice. Two research teams (Stelloo E with his cooperators and Talhouk A with his cooperators) tested more pragmatic methods that identify distinct subgroups with a prognostic based on the results of TCGA. *Trans*PORTEC was a translational research in high-risk endometrial cancer (Stelloo et al., 2015). They identified four EC subgroups similar like results of TCGA, and highlighted the potential of the molecular classification to refine and further individualize patients’ risk stratification. Talhouk et al. (2015, 2017) developed and validated a Proactive Molecular Risk Classifier for Endometrial Cancer (ProMisE) groups in a new, large cohort of ECs, which classified Talhouk the EC into four subgroups of including POLE-mutated (POLE-mt), mismatch repair-deficient (MMR-d), p53-abnormal (p53-abn), p53-wild-type (p53-wt). ProMisE group contained the different molecular signatures compared to other methods, and could improve the ability to discern outcomes when it combined with the addition of select parameters. Moreover, a series of studies were conducted to analysis the clinical and histopathological characterization of ProMisE molecular groups, and revealed that ProMisE groups could identify different phenotypes of patients, and a great percentage of patients are currently under- or over treated (Raffone et al., 2020, 2021a). They also found that tumor-infiltrating lymphocytes might be considered in an integrate algorithm to identify POLE-mutated ECs when sequencing is unavailable (Raffone et al., 2021b). These results might lay the groundwork for future clinical translation of these stratification methods.

Compared the clinical and histopathological characteristics between the two subtypes in our study and the previously ProMisE and *Trans*PORTEC, we found that there were obvious biologic relevance of these molecular features among these three classifications. For example, subtype I cases identified in our study had a high percentage of stage I and grade I–II, and less tumor invasion with favorite prognosis. These characteristics were similar to these EC cases in p53wt group identified in ProMisE, which also had the high prevalence of stage I and low-grade and low prevalence of lymphovascular space invasion with the good to moderate prognosis (Raffone et al., 2020, 2021a). While the subtype II cases in our study had an analogous molecular features with the group5 EC cases named “TP53 mutated/Non-homologous End-Joining positive” group defined in *Trans*PORTEC (Auguste et al., 2018), as both of them had high expression of DNA damage and PARP-1 expression with the worst prognosis.

Genes up-regulated in subtype I EAC were found to be involved in the immune response, defense response, cell motion, and cell motility. For example, *CRISP3* has been discovered in human neutrophilic granulocytes and has a role in the innate host defense (Udby et al., 2002). C4a is a small protein released from complement component C4, which is an important constituent of innate immune surveillance (Wang et al., 2017). *SCNNIB* encodes a component of a sodium channel that controls fluid and electrolyte transport across epithelia in a diverse range of organs, and it induces cell apoptosis and cell cycle arrest in gastric cancer (Qian et al., 2017).

Genes up-regulated in subtype II EAC were found to be involved in the cell cycle, cell division, DNA replication, and RNA processing. For example, *ADCY3* is a membrane-associated protein that is widely expressed in human tissues, and it exhibits tumor-promoting effects via the cAMP/PKA/CREB pathway (Hong et al., 2013). *MSH2* and *MSH6* are components of the post-replicative DNA mismatch repair (MMR) system that bind to DNA mismatches, thereby initiating DNA repair (Seifert et al., 2008). *MSH2*, *MSH6*, *MLH1*, and *PMS2* are deficient mismatch repair (dMMR) proteins up-regulated in Lynch syndrome and ovarian endometrioid carcinoma (Rambau et al., 2016). Lynch Syndrome is a highly penetrant, autosomal dominant cancer predisposition syndrome caused by mutation of mismatch repair genes, specifically MLH1, MSH2, MSH6, or PMS2. The probable rates of radical changes in MSH2, MLH1, and MSH6 were 50–66%, 24–40%, and 10–13% in endometrial carcinoma associated with Lynch syndrome (Bonadona et al., 2011). With the finding that mismatch repair proteins such as *MSH2* and *MSH6* were identified as more common in subtype II tumors, it may be hypothesized that the Lynch syndrome-related endometrial cancer may be mainly subtype II EAC. *PSMD2*, *PSMD3*, *PSMD7*, and *PSMD8* are components of the 26S proteasome, a multiprotein complex involved in the ATP-dependent degradation of ubiquitinated proteins. This complex plays a key role in the maintenance of protein homeostasis by removing misfolded or damaged proteins (which can impair cellular functions) and by removing proteins that are no longer required (Kanayama et al., 1992). *PSMD2* is significantly dysregulated in breast cancer and associated with poor prognosis (Li et al., 2018).

Currently, the main treatments for EAC are surgery, radiotherapy, chemotherapy, or combinations of these (Creasman et al., 2006). Although many EAC patients can be cured by surgery or radiotherapy plus chemotherapy, about 15–20% of EAC patients with no signs of locally advanced or metastatic disease in the primary treatment period experience a recurrence, with limited treatment response (Engelsen et al., 2009). Therefore, molecular subtyping of EAC not only stratifies the EAC population into subgroups with different risks, but also provides insights into the development of targeted therapies. We found that there are a number of known potential therapeutic targets up-regulated in subtypes I and II, respectively, which have also been identified as potential targets in *Trans*PORTEC research (Stelloo et al., 2015).

It is noteworthy that *PIK3R1* is up-regulated in subtype I EAC, indicating PI3K/AKT/MTOR inhibitors may be a potential therapeutic target for cases of subtype I EAC. The mutation of PIK3R1 has been found in colorectal, breast, and ovarian cancer (Philp et al., 2001), and is also a potential therapeutic target in glioblastoma multiforme, as it regulates tumor cell growth and motility (Weber et al., 2011). In breast and ovarian cancer patients, the inhibition of the PI3K/AKT/MTOR pathway with PIK3R1 inhibitors (temsirolimus, ridaforolimus, everolimus) have been shown to satisfactory clinical outcomes of patients with endometrial cancer in Phase II trials (Slomovitz et al., 2010, 2015; Oza et al., 2011). Everolimus plus letrozole (aromatase inhibitor) results in a clinical benefit rate (CBR) of 40% in patients with recurrent EC (Slomovitz et al., 2015). In addition, progestin-based hormonal therapy has long been used to treat the hyperestrogenism associated with endometrial hyperplasia and carcinoma. The Gynecologic Oncology Group (GOG) study revealed that high-dose medroxyprogesterone acetate in treatment of advanced and recurrent endometrial cancer showed a 24% clinical RR (Lentz et al., 1996), and the combination of tamoxifen and medroxyprogesterone acetate could be an active treatment for advanced or recurrent endometrial carcinoma with clinical RRs of 27–33% (Whitney et al., 2004).

Subtype II EACs exhibited up-regulated cell cycle-related genes including *CDKN1B*, *CCNE1*, *CDK4*, *EZH2*, *CRLK*, *BRCA2*, *BRCA1*, *CDKN1B*, *CCNE1*, and *CDK4*. The functions of the proteins encoded by these genes can be inhibited by the CDK4/6 inhibitors ribociclib and abemaciclib. These inhibitors have been used to treat many cancers including colorectal cancer, breast cancer, non-small cell lung cancer, and melanoma (Patnaik et al., 2016), and a phase II study is underway to evaluate effect of letrozole and ribociclib (CDK4/6 inhibitor) in patients with relapsed ER-positive endometrial cancer (NCT02657928). Alterations with the RAS/RAF/MEK pathway were also enriched in Subtype II EACs. A recent GOG phase II trial was undertaken to explore the role of MEK inhibitor (selumetinib) in recurrent endometrial cancer, and revealed that the clinical responses were 6 and 26% with stable disease (Coleman et al., 2015). In addition, EZH2 is upregulated in endometrial cancer, and overexpression of EZH2 is significantly associated with high histologic grade, lymph node metastasis, and cervical involvement, which could serve as potential therapeutic targets for subtype II EAC patients (Zhou et al., 2013; Jia et al., 2014). Similarly, PTEN mutant endometrial cancer cell lines have been reported to have increased sensitivity to PARP inhibitors. Ongoing studies evaluating the use of PARP inhibition in endometrial cancer are investigating biomarker response and resistance (NCT02208375; NCT02127151).

In conclusion, we defined two clinically relevant EAC molecular subtypes, which were identified in two independent cohorts. Our study provides new insights to explore the mechanisms underlying the tumorigenesis and progression of EAC and offers opportunities to develop subtype-specific diagnostic biomarkers and targeted therapeutic treatments.

## Data Availability Statement

The original contributions presented in the study are included in the article/Supplementary Material, further inquiries can be directed to the corresponding author/s.

## Author Contributions

JL, RG, and QK were responsible for the study concept and design. JL and SY collected the data, performed the analyses, and drafted the manuscript. XG, FW, and GZ performed the analyses and contributed to manuscript writing. All authors edited and approved the final manuscript.

## Conflict of Interest

The authors declare that the research was conducted in the absence of any commercial or financial relationships that could be construed as a potential conflict of interest.

## Footnotes

1 https://david.ncifcrf.gov/

2 https://www.broadinstitute.org/cancer/cga/target

## Supplementary Material

Supplementary Figure 1 The flowchart of identification of distinct molecular subtypes of Endometrioid Adenocarcinoma.
