# Supplementary figures and images for: Identification of Distinct Molecular Subtypes of Endometrioid Adenocarcinoma
Source: Front Genet. 2021 Jul 21;12:568779. doi: 10.3389/fgene.2021.568779 (PMC8334731; doi:10.3389/fgene.2021.568779)

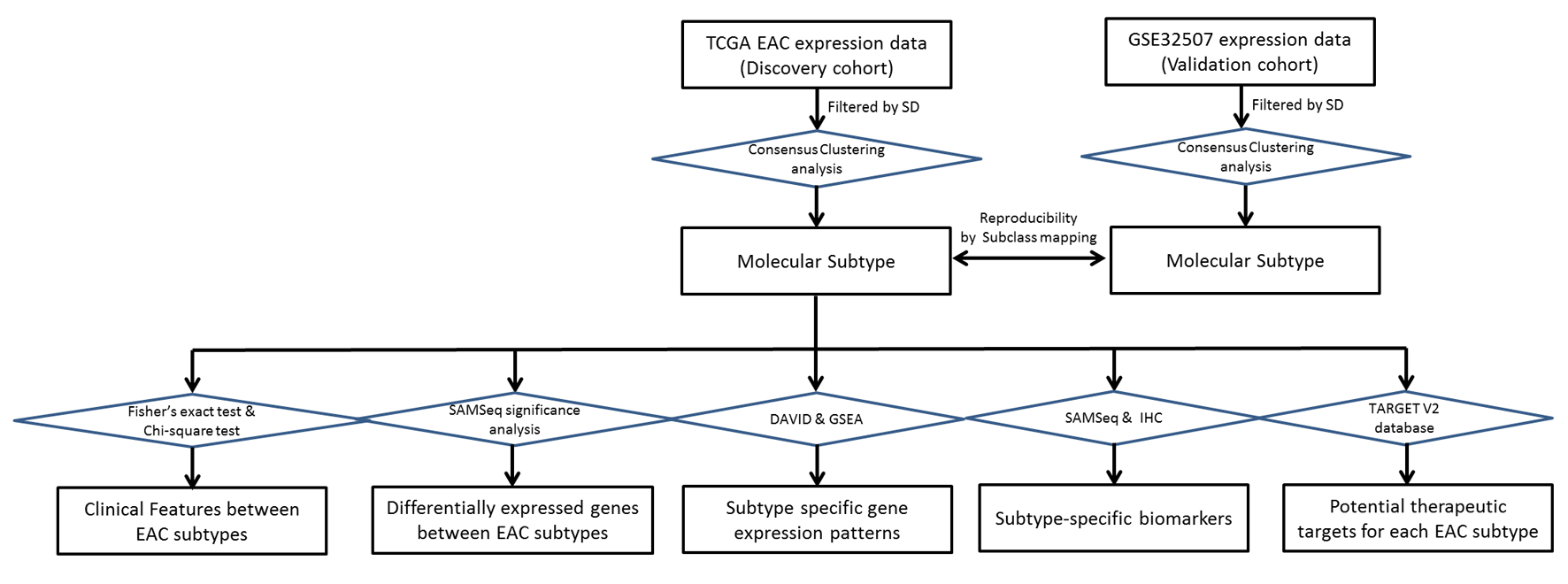

Supplement: Supplementary Figure 1 — The flowchart of identification of distinct molecular subtypes of Endometrioid Adenocarcinoma. [file Image_1.TIF]
